# Maternal effects on offspring size and number in mosquitofish, *Gambusia holbrooki*

**DOI:** 10.1002/ece3.1577

**Published:** 2015-07-03

**Authors:** Rose E O’Dea, Regina Vega-Trejo, Megan L Head, Michael D Jennions

**Affiliations:** Evolution, Ecology and Genetics, Research School of Biology, Australian National UniversityCanberra, ACT, Australia

**Keywords:** Maternal investment, nonlinear relationship, optimality model, trade-off

## Abstract

Given a trade-off between offspring size and number, all mothers are predicted to produce the same optimal-sized offspring in a given environment. In many species, however, larger and/or older mothers produce bigger offspring. There are several hypotheses to explain this but they lack strong empirical support. In organisms with indeterminate growth, there is the additional problem that maternal size and age are positively correlated, so what are their relative roles in determining offspring size? To investigate this, we measured the natural relationship between maternal and offspring size in a wild population of *Gambusia holbrooki* (eastern mosquitofish), and experimentally disentangled the effects of maternal age and size on offspring size in the laboratory. In combination, our data indicate that the relationship between maternal and offspring size is nonlinear. Small mothers seem to produce larger than average offspring due to integer effects associated with very small broods. For extremely large mothers, which were only sampled in our wild data, these larger than average offspring may result from greater maternal resources or age effects. However, maternal age had no effect on offspring size or number in the laboratory experiment. Our results highlight the importance of sampling the full size–range of mothers when investigating maternal effects on offspring size. They also point to the difficulty of experimentally manipulating maternal size, because any change in size is invariably associated with a change in at least one factor affecting growth (be it temperature, food availability, or density) that might also have an indirect effect on offspring size.

## Introduction

Maternal fitness depends on how many offspring are produced and how well these offspring survive and reproduce (i.e., their reproductive value). Mothers have finite resources to invest in reproduction so they face a trade-off between offspring size and fecundity (Roff 1983; Pollux and Reznick 2011). But what is the optimal offspring size? From a mother’s perspective, larger offspring survive better than smaller ones (Einum and Fleming 1999; Johnston and Leggett 2002; Kuijper and Johnstone 2013; Omkar and Afaq 2013), but the size-fecundity trade-off counters an unfettered increase in offspring size (Trivers 1974). From the offspring’s perspective, being as large as possible at birth is best (Blanckenhorn 2000; Rollinson and Hutchings 2013). It is, however, generally assumed that mothers control offspring size and have the upper hand in any parent–offspring conflict, especially when there is placental or maternal care (Steiger 2013). Most theoretical models therefore assume that offspring size maximizes maternal fitness (Marshall and Keough 2008).

The 1970s saw the development of a landmark model to determine the optimal maternal solution to the size-fecundity trade-off (Smith and Fretwell 1974). Smith and Fretwell modeled offspring fitness as a function with diminishing returns. That is, offspring fitness increases as mothers invest more, but the marginal rate of increase slows and approaches zero at the point where all resources are invested into one individual. The optimal offspring size occurs at the point of maximum returns on the offspring fitness curve. A shallower curve (i.e., smaller marginal gains) reflects a harsher environment, in which offspring need to be bigger to survive (Einum and Fleming 1999; Marshall et al. 2010). For example, the seed beetle *Stator limbatus* changes the size of its eggs to suit its host plant (Fox et al. 1997). Mothers produce larger eggs on plants that have lower larval survival, and therefore lay fewer eggs than when they lay eggs in a more benign environment.

Real-life patterns of offspring investment often defy the predictions of the optimality model for offspring size (Hutchings 1991; Marshall et al. 2010; Kindsvater et al. 2011). The model predicts that within a population, all mothers in the same environment should produce the same-sized offspring. Mothers with more resources should simply produce additional optimal-sized offspring. Maternal size is predicted to be positively correlated with offspring number, but uncorrelated with offspring size. A recent meta-analysis of 241 species from a wide range of taxa found, however, that maternal size tends to be positively correlated with both offspring number and size (Lim et al. 2014). While positive correlations between traits that are traded-off against one another can be an outcome of resource heterogeneity within a population (van Noordwijk and de Jong 1986), it is unclear why larger (resource-rich) mothers increase offspring size rather than offspring number. Furthermore, it has also been noted that older mothers produce larger offspring (Ribi and Gebhardt 1986; Glazier 1992; Ito 1997; Berkeley et al. 2004). Such maternal age effects could be frequently overlooked and attributed to maternal size due to a positive size–age correlation in many taxa (i.e., those with indeterminate growth) (Marshall et al. 2010).

There are several competing hypotheses to explain why maternal size and/or age affects (or is positively correlated with) offspring size (Marshall and Keough 2008). Most theoretical models focus on maternal size effects. For example, one of the earliest ideas was that the higher fecundity of larger mothers induces sibling competition, and that therefore their offspring need to be larger to compensate for this effect (Parker and Begon 1986). This explanation is more likely to apply in species when offspring do not disperse as juveniles (Kindsvater et al. 2012). A similar argument applies to a maternal age effect: life-history theory predicts that mothers face a trade-off between current and future reproduction (Williams 1966). If older mothers have a decreased likelihood of future reproduction (i.e., senescence), they are predicted to increase their investment in the current reproductive attempt (Pianka and Parker 1975). This may be accompanied by a concurrent increase in offspring size, to compensate for density-dependent sibling competition (Benton et al. 2008). Another model for a maternal age effect on offspring size asserts that if decreased reproductive effort increases longevity, then it is more advantageous for young mothers to reduce offspring size than number (assuming that it costs more to sacrifice fecundity; that is, lower fecundity has a stronger effect on fitness than does producing smaller sized offspring) (Kindsvater et al. 2012). In contrast, if older mothers have a lower expectation of future survival, they are predicted to produce the optimal offspring size irrespective of the associated survival risks.

Hypotheses for why larger or older mothers produce larger offspring generally lack robust corroborating empirical evidence (Marshall and Keough 2008). A key problem is identifying whether it is maternal age or size that is important, as these two factors are often correlated (Marshall et al. 2010). There are some studies in organisms with determinate growth that separate the effects of maternal age and size statistically (e.g., in the wandering albatross (*Diomedea exulans*) (Blanchard et al. 2007) and the wood duck (*Aix sponsa*) (Hepp and Kennamer 1993) maternal size, but not age, was correlated with offspring size). Experimental studies, however, are crucial to understand variation in life-history trade-offs, and how parent–offspring conflict over resource allocation into offspring size is resolved. In this study we use a species with indeterminate growth to experimentally tease apart maternal age and size to test their causal effects on offspring size.

Here, we investigate the effects of maternal age and size on offspring size and number in an organism with indeterminate growth, *Gambusia holbrooki* (eastern mosquitofish), a poeciliid fish with no postnatal parental care (Evans et al. 2011). This implies that mothers are under strong selection to produce optimal-sized offspring, because if they produce the “wrong-” sized offspring they cannot compensate by subsequently adjusting levels of care (Marshall et al. 2010; Steiger 2013). Sexually mature female *G. holbrooki* exhibit large size variation, ranging from 20 to 60 mm in standard length (SL) (Pyke 2005), which provides ample scope to study the effects of maternal size on offspring size. Their short life spans (generally <1 year in the wild) and brief breeding season in our study population (November to March) also mean that biologically significant age differences between *G. holbrooki* can readily be generated (Cabral and Marques 1999; Pérez-Bote and López 2005).

We investigate the relationship between maternal size/age, offspring size, and offspring number in a wild population of *G. holbrooki* and show that larger/older mothers have more and bigger offspring than smaller/younger mothers. We then experimentally manipulate the size and age of female fish in the laboratory to investigate the independent contributions of maternal size and age to this relationship. Our findings, and their interpretation, highlight the challenges associated with determining the factors causally responsible for variation in offspring size.

## Materials and methods

### Field methods

In January 2014, we captured 70 pregnant *G. holbrooki* from a pond in Canberra, Australia (35°18′27″S°149°07′27.9″E). To identify pregnant *G. holbrooki,* we indiscriminately caught fish with a hand net and deposited them into containers containing pond water. Pregnant females were identified as those with swollen abdomens. In the laboratory, we housed pregnant *G. holbrooki* individually in 1 L aquaria. Each tank contained a mesh divider, creating refugia for fry. We checked tanks for fry twice daily for 2 weeks after capture. Four females who did not give birth were discarded. We euthanized females after they had given birth and recorded their SL (SL = snout tip to base of caudal fin) (mm) by photographing them next to a scale ruler. We did not return fish to the wild because *G. holbrooki* are an invasive species in Australia (Macdonald et al. 2012) and it is illegal to do so. The size range of females that gave birth was 25.28–47.61 mm in length (*n* = 66; mean = 32.90; standard deviation [SD] = 6.10).

To measure the SL of fry, we took an overhead photograph of individual fry in water (5 mm deep) held in a small transparent container, placed atop 1 mm scale graph paper. The resultant images were analyzed using *Image J* (Schneider et al. 2012). Mean offspring size per female ranged from 6.68 to 7.82 mm (*n* = 66; mean = 7.22; SD = 0.29). We measured the SL of up to 10 fry per brood at birth (randomly selected from the tank they were born into), and noted the brood size. Brood size ranged from 1 to 104 (*n* = 66; mean = 23.74; SD = 19.93). We chose to measure a maximum of 10 fry per brood to strike a balance between obtaining an accurate estimate of the average offspring size within a brood, and obtaining comparable information on within-brood offspring size variation. Earlier pilot studies showed that there was very low variability in offspring size within a brood.

### Experimental manipulation of maternal size and age

**Figure 1:**
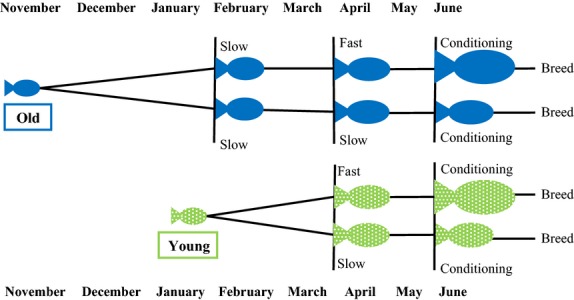
Experimental design to obtain four groups of females (Young/Old, Small/Large). Slow-growth conditions were as follows: 19°C, higher densities and fed once daily. Fast-growth conditions were as follows: 28°C, lower densities and fed ad libitum. All mothers were placed in a favorable (“conditioning”) environment for 1 month prior to breeding.

To disentangle the effects of maternal size and age on offspring size, we used the daughters of wild-caught *G. holbrooki* in laboratory breeding experiments. We had four cohorts of females: Large/Old (*n* = 56), Large/Young (*n* = 68), Small/Old (*n* = 72), and Small/Young (*n* = 84). In brief, we slowed the growth of the first, older cohort until the second, younger cohort caught up in size. We then split each cohort into two groups: One was placed in fast-growing conditions to become large and the other into slow-growing conditions to stay relatively small (Fig.1). In the fast-growing conditions, we kept fish at low densities (initially 20 individuals per 90 L, reduced over time), at 28°C. In addition, fish were fed both *Artemia nauplii* and commercial fish flakes multiple times per day. Slow-growing conditions consisted of fish being kept at higher densities (eight individuals per 6.5 L), at a cooler temperature (19°C), where we fed them once daily, on a diet of *A. nauplii* (Vondracek et al. 1988; Pérez-Bote and López 2005). The range in length (in mm) of the females that gave birth was as follows: Large/Old: 33.00–37.82 (*n* = 23; mean = 35.40; SD = 1.24), Large/Young: 30.55–38.35 (*n* = 42; mean = 34.57; SD = 1.68), Small/Old: 23.77–29.52 (*n* = 36; mean = 26.78; SD = 1.40), Small/Young: 24.19–28.50 (*n* = 39; mean = 26.41; SD = 1.21).

Our laboratory-reared mothers were born in captivity in either November 2013 (Old) or January 2014 (Young). They were initially maintained at 28°C (five individuals per 2.5 L) and separated from males as soon as sexable (from 3 weeks of age onwards). To create similar sized individuals in the young and old-age classes, the Old females were kept in the slow-growing conditions for 2 months longer than the Young females (February to April, Fig.1). This allowed the Young females to catch up in size to Old females by April (see Results).

In April 2014, we took half of the Old females, and half of the Young females, and housed them in fast-growing conditions, while the other half was housed in slow-growing conditions. To monitor the efficiency of our experimental treatments, we measured the size of a subsample of fish weekly. We marked 10 fish from each of the four groups (Large/Young, Large/Old, Small/Young, Small/Old) with fluorescent elastomer (Northwest Marine Technology, USA) injected subcutaneously behind the caudal fin.

To ensure females were in reproductive condition, they all underwent a conditioning period before breeding. They were kept at 28°C and fed a diet of *A. nauplii* and commercial fish flakes. The small treatment fish were, however, kept at higher densities than the large treatment fish (60 fish per 60 L compared to 10 fish per 90 L) to maintain the size difference during this time. The conditioning period lasted for 1 month, after which all females were reproductive (as indicated by two black spots near their genital opening; Pyke 2005). It is unavoidable that a mother’s rearing conditions might affect offspring size due to her diet or rearing temperature rather than her size per se, but the conditioning period reduced the possibility of direct, short-term effects of maternal rearing conditions on offspring size. (It is obviously impossible to change a female’s size and control for age without changing some aspect of her rearing conditions).

### Breeding design

Females were set up to breed in June 2014 when the old and young cohorts were 7 and 5 months old, respectively. We placed one male with four females in 6.5-L aquaria. Males were first generation laboratory stock. After 1 week, we removed the male and separated the females into individual 1-L aquaria containing a mesh divider.

Three weeks after the male was first introduced (the minimum *G. holbrooki* gestation period; Pérez-Bote and López 2005), we began to check for fry twice daily. We measured the SL of the fry on the day they were born, and the SL of the mothers the following day, as per the protocol for wild-caught females. We discarded any females that had not produced offspring within 48 days of being with a male. A total of 142 of 280 females bred within the allotted period (the standard mean success rate in our laboratory). The breeding success of both Large and Small females was approximately equal (Large/Old: 41.1%, Large/Young: 61.8%, Small/Old: 51.4%, Small/Young: 47.6%), indicating that the conditioning period succeeded in controlling for the short-term condition of Large versus Small females.

All mothers and fry were given a unique ID before being photographed, to ensure that we made measurements blind to their treatment group. For analyses, we used the average size of offspring in a brood. Offspring size within broods was repeatable within each of the four treatment groups (intraclass correlations: Large/Old: *r *=* *0.50, *n* = 207 fry, 23 mothers; Large/Young: *r *=* *0.55, *n* = 380 fry, 42 mothers; Small/Old: *r *=* *0.59, *n* = 234 fry, 36 mothers; Small/Young: *r *=* *0.85, *n* = 227 fry, 39 mothers; all *P* < 0.01). There was no evidence that within-brood variation in offspring size differed as a result of maternal size or age (Size: *F*_1,130_ = 0.08, *P *=* *0.78, Age: *F*_1,130_ = 1.86, *P *=* *0.18; for analysis details see below). The range in length (mm) of the fry for the four groups of mothers was as follows: Large/Old: 6.84–7.58 (*n* = 23; mean = 7.30; SD = 0.21), Large/Young: 6.96–7.98 (*n* = 42; mean = 7.35; SD = 0.22), Small/Old: 6.87–7.95 (*n* = 36; mean = 7.42; SD = 0.27), and Small/Young: 6.51–8.84 (*n* = 39; mean = 7.48; SD = 0.49).

Before analysis, we removed two outliers as: (i) one brood consisted of a single offspring more than six standard deviations larger than the mean offspring size. It is highly likely that we overlooked its existence on its day of birth so that it was already at least 1 day old and (ii) one “Old/Small” mother was in the same size range as the “Old/Large” mothers, indicating that her response to the size manipulation treatment was atypical (including her in our analysis did not qualitatively alter our results).

### Statistical analyses

To examine the effects of maternal size and age on offspring number and size, we performed a multivariate analysis of variance (MANOVA). We specified offspring size and number as dependent variables, and Age (Old/Young) and Size class (Large/Small) as fixed factors. We included the interaction term.

To examine the effects of maternal size and/or age on offspring size from wild-caught females, we performed partial correlation analyses to examine the relationships between maternal size, offspring size, and offspring number. We used the false discovery rate method to correct the *P*-values for multiple comparisons.

For direct comparison with wild-caught females, we also conducted bivariate correlations on the experimental females to examine the relationships between maternal size, offspring number and offspring size, pooling across maternal age and size classes.

Our laboratory experiment yielded different results to those for wild-caught females. To examine possible reasons for this, we conducted additional exploratory analyses. First, to examine why the relationships between maternal and offspring size, as well as offspring number and offspring size, differed between experimental and wild-caught mothers, we repeated the original analyses on wild fish (partial correlations) but only included mothers within the size range generated in the laboratory (i.e., mothers <40 mm).

To investigate whether the relationship between offspring number and size differed between large and small mothers, we ran separate analysis of covariance (ANCOVA) for the experimental and wild-caught mothers, with offspring size as the dependent variable, maternal size and age (laboratory only) as fixed factors, and standardized offspring number (standardized so that mean = 0 and SD = 1; see Schielzeth 2010) as a covariate. We included the interaction term in the final model, as we were interested in any difference in the slope of the relationship. To generate comparable size classes for the wild-caught females, we classified them using the size ranges for small and large class laboratory-reared mothers (Small: 23.5–29.5 mm; Large: 30.5–40.0 mm).

To test whether the age or size treatments affected within-brood heterogeneity in offspring size, we ran an ANCOVA on offspring size variance for each brood, with age and size as fixed factors, and mean offspring size as a covariate.

For all models, we checked standardized residuals for normality. Where log-transformation of variables improved the normality of residuals we present these results. Effect sizes (Cohen’s *d*) were calculated from partial eta squared values. We ran analyses with SPSS v. 22.0 (Armonk, NY, USA).

## Results

### Do larger mothers produce more and bigger offspring in the wild?

**Figure 2:**
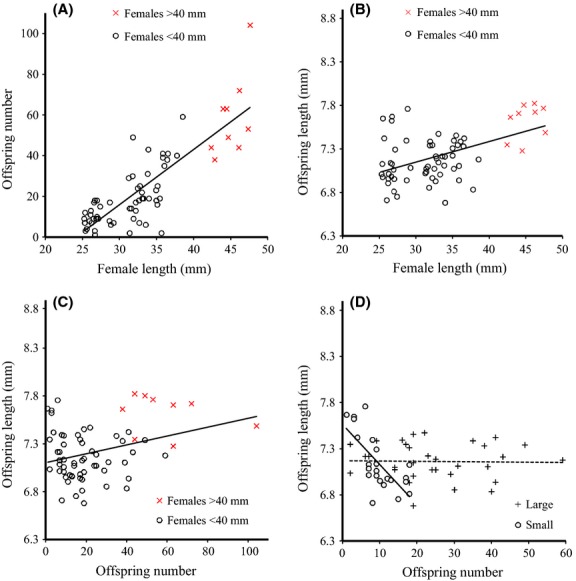
Wild population results. Analysis was on log-transformed data for panels B, C, and D. (A) The relationship between maternal size and number of offspring (*y* = −65.48 + 2.71*x*, *R*^2^ = 0.69, *P *<* *0.001); (B) the relationship between mother’s size and offspring size (*y* = 0.70 + 0.11*x*, *R*^2^ = 0.22, *P *<* *0.001); (C) the relationship between the number of offspring in a brood and their average size (*y* = 0.85 + 3.52*10^−3^*x*, *R*^2^ = 0.01, *P *=* *0.50); (D) the relationship between the number of offspring in a brood and their average size for mothers assigned to the Large and Small categories. There was a strong negative correlation between the number of offspring in a brood and their average size among small females. The regression lines are shown (Large: *y* = 0.86 − 2.34*10^−3^*x*, *R*^2^ = 0.01, *P *=* *0.71; Small: *y* = 0.90 − 0.05*x*, *R*^2^ = 0.58, *P *<* *0.001).

Larger wild-caught mothers had more and bigger offspring than smaller wild-caught mothers (offspring number: *r *=* *0.82, 95% CI [0.72, 0.88], *n* = 66, *P *<* *0.001, Fig.2A; offspring size: *r *=* *0.59, 95% CI [0.41, 0.73], *n* = 66, *P *<* *0.001; Fig.2B). Larger broods were comprised of smaller offspring (*r* = −0.41, 95% CI [−0.59, −0.19], *n* = 66, *P *<* *0.001) (although with a standard bivariate correlation, which does not control for maternal size, there was no relationship between offspring number and offspring size for wild-caught mothers (*r *=* *0.08, 95% CI [−0.16, 0.32], *n* = 66, *P *=* *0.502; Fig.2C).

### How do maternal size and age affect the number and size of offspring?

**Figure 3:**
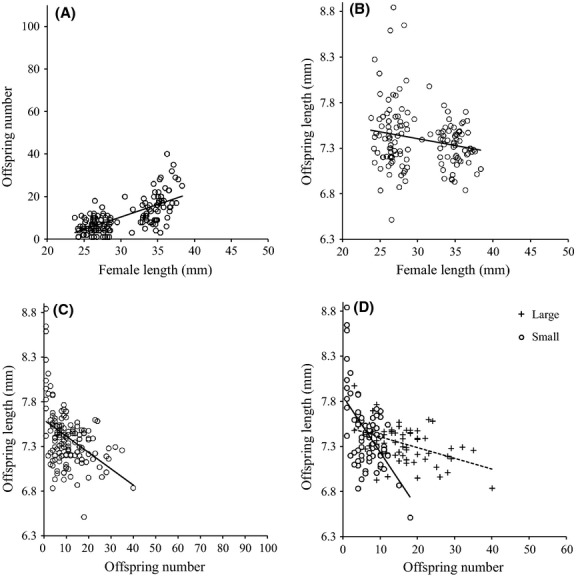
Experimental results for laboratory-reared fish. Analysis was on log-transformed data for panels B, C, and D. (A) The relationship between maternal size and number of offspring (*y* = −24.96 + 1.18*x*, *R*^2^ = 0.46, *P *<* *0.001); (B) the relationship between mother’s size and offspring size (*y* = 0.95 − 0.06*x*, *R*^2^ = 0.06, *P *=* *0.02); (C) the relationship between the number of offspring in a brood and their average size (*y* = 7.60 − 0.02*x*, *R*^2^ = 0.17, *P *<* *0001); (D) there was a stronger negative correlation between the number of offspring in a brood and their average size among small females (Large: *y* = 0.89 − 0.02*x*, *R*^2^ = 0.19, *P *<* *0.001; Small: *y* = 0.90 − 0.04*x*, *R*^2^ = 0.42, *P *<* *0.001).

Maternal size class, but not age class, affected offspring size (Table1). However, in contrast to the pattern seen for wild-caught mothers, the larger laboratory-reared mothers actually produced smaller offspring (*r *=* *−0.19, n = 140, *P *=* *0.024; Fig.3B).

**Table 1:** Multivariate analysis of variance (MANOVA) test of between-subject results

Source	Type III sum of squares	df	Mean square	*F*	*d*	*P*
Corrected model						
Offspring number	3436.26	3	1145.42	33.80	1.73	0.000
Offspring size	0.60	3	0.20	1.83	0.40	0.144
Intercept						
Offspring number	17721.77	1	17721.77	522.96	3.92	0.000
Offspring size	7236.54	1	7236.54	66479.16	44.22	0.000
Age						
Offspring number	225.55	1	225.55	6.66	0.44	0.011
Offspring size	0.12	1	0.12	1.08	0.18	0.300
Size						
Offspring number	3391.71	1	3391.71	100.09	1.72	**0.000**
Offspring size	0.51	1	0.51	4.67	0.37	**0.033**
Age^*^size						
Offspring number	140.88	1	140.88	4.16	0.35	**0.043**
Offspring size	0.00	1	0.00	0.00	0.01	0.944
Error						
Offspring number	4608.67	136	33.89			
Offspring size	14.80	136	0.11			
Total						
Number of offspring	24613.00	140				
Offspring size	7676.89	140				
Corrected total						
Number of offspring	8044.94	139				
Offspring size	15.40	139				

Age = age class (Old/Young); Size = Size Class (Large/Small).

Values in bold represent significant *P-*values.

**Figure 4:**
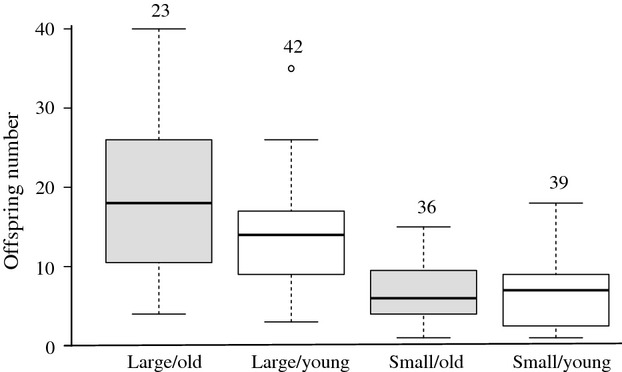
Box-and-whisker plot of the two-way interaction between female size class and female age class on offspring number. Open circles represent outliers. Sample sizes are displayed above the whiskers.

There was a significant interaction between the size class and age class of laboratory-reared mothers that affected brood size (Table1). Larger mothers had significantly more offspring (*r *=* *0.68, *n* = 140, *P *<* *0.001; Fig.4). For the large size class, old mothers produced significantly more offspring than young mothers, but there was no effect of age on fecundity for the small size class mothers (Fig.4).

**Figure 5:**
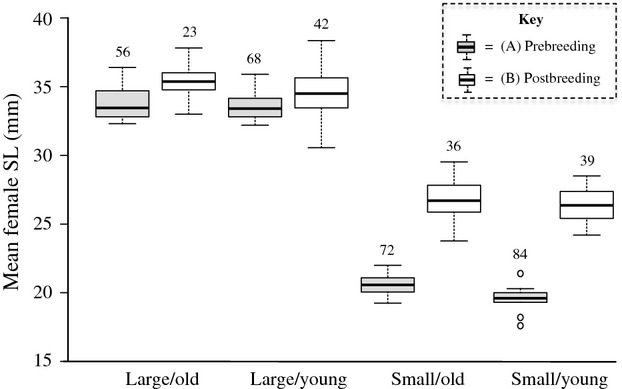
Female sizes measured (A) 7 days prior to being exposed to a male on 24th June 2014 (gray boxplots); (B) that eventually gave birth between 15th July and 11th August 2014 (white boxplots). The Small females continued to grow until they gave birth, reducing the ultimate size difference between the large and small size classes, and the Old females were slightly larger than the Young females. Open circles represent outliers. The number of females in each group is displayed above the whiskers. (Note that not all females bred, so this also affects the size differences between treatment groups for postbreeding females).

The effect of age class on offspring number might have been due to a small difference in the actual mean size of old and young mothers in the large size class (Fig.5). To test for this, we restricted our analysis to large size-classes females and ran an ANCOVA with offspring number as a response variable, age class as a fixed factor, and standardized female SL (again, see Schielzeth 2010) as a covariate. Female size (SL) was now the only significant predictor of offspring number (Age class: *F*_1, 62_ = 1.17, *d *=* *0.28, *P *=* *0.284; Female SL: *F*_1, 62_ = 19.13, *d *=* *1.12, *P *<* *0.001; Age class*Female SL: *F*_1, 62_ = 3.13, *d *=* *0.45, *P *=* *0.082) despite the narrow range in female size (narrow because all females were in the same (i.e., large) size class).

Overall, there was a negative relationship between offspring number and size (*r* = −0.57, *n* = 140, *P* < 0.001); however, a significant interaction shows that this relationship was far stronger for small size class mothers than it was for large size class mothers (size class × offspring number interaction: *F*_1,133_ = 3.97, *d *=* *0.35, *P *=* *0.048) (Fig.3D). In contrast, there was no difference in the size–number relationship between young and old-age class mothers (*F*_1,133_ = 2.79, *d *=* *0.29, *P *=* *0.097).

### Why is there a difference between wild-caught mothers and laboratory-reared mothers?

The positive relationship between maternal and offspring size in wild-caught mothers was much weaker when we restricted the data to the maternal size range in our laboratory breeding experiment (SL <40 mm) (*r *=* *0.34, 95% CI [0.09, 0.55], *n* = 57, *P *=* *0.01). As before there was still a significant negative correlation between offspring number and size (*r* = −0.44, 95% CI [−0.63, −0.20], *n* = 57, *P *=* *0.021), but this correlation remained even when female size was not controlled in the analysis (*r* = −0.30, 95% CI [−0.52, −0.05], *n* = 57, *P *=* *0.02). When we categorized wild-caught females into the small and large size classes, it was clear that, as for the laboratory-reared mothers, smaller mothers had a steeper reduction in offspring size with increasing offspring number (size class*offspring number: *F*_1, 53_ = 17.93, *d *=* *1.15, *P *<* *0.001; Fig.2D).

## Discussion

Theory predicts that mothers should produce offspring of an optimal size and that mothers with more resources should produce more offspring rather than larger offspring (Smith and Fretwell 1974). This should create a positive relationship between maternal size and offspring number, but not between maternal size and offspring size. Our results indicate that these predictions hold for intermediate-sized mothers, but breakdown at extreme sizes. These findings highlight the importance of incorporating nonlinear relationships between life-history traits into predictions about optimal maternal allocation.

### Relationship between maternal size/age and offspring size

We experimentally disentangled the effects of maternal size and age on offspring size in the laboratory. Maternal size, but not age, affected offspring size. Unexpectedly, however, larger mothers produced smaller offspring. This was opposite to the relationship seen in the wild. Larger wild-caught mothers produced larger offspring, as seen in many other species (meta-analysis: Lim et al. 2014), including some poeciliid fish (Benejam et al. 2009; Swenton and Kodric-Brown 2012). Additional exploratory analyses revealed that the very largest wild *G. holbrooki* drove this trend. Wild-caught mothers in the size range of our laboratory-reared mothers (<40 mm SL) showed no relationship between maternal and offspring size. Our results suggest that the relationship between maternal and offspring size in *G. holbrooki* is nonlinear: Both small and large mothers have larger offspring than medium-sized mothers. This may explain the inconsistent results reported in the literature for the relationship between maternal and offspring size among *Gambusia*, where there is evidence of negative (Lim et al. 2014 – unpublished data cited in the meta-analysis digital repository), no (*Gambusia affinis*: Swenton and Kodric-Brown 2012), and positive correlations (*G. holbrooki*: Benejam et al. 2009; *Gambusia nobili*: Swenton and Kodric-Brown 2012). If the relationship between maternal and offspring size is nonlinear, then it is possible to obtain each of these results by sampling a subset of the full maternal size–range. For example, we would have found a negative relationship between maternal and offspring size if we had failed to sample very large females, a null relationship if we only sampled medium-sized females, and a positive relationship if we only sampled medium- and large-sized females.

The difference in the strength of the negative relationship between maternal and offspring size in laboratory-reared females of different size classes might be due to integer effects (and the fact that small females produce small broods). Because a mother’s number of offspring must be an integer (they cannot produce a fraction of an offspring), the optimality model for offspring size fails at small brood sizes (Charnov and Downhower 1995; West et al. 2001). For example, if the total amount of resources a mother has to invest is 1.2 times the optimal level of investment per offspring, she can either produce two small or one large offspring (Charnov et al. 1995). If the fitness cost of producing offspring smaller than the optimal size is sufficiently high, then smaller broods will tend to have larger than average offspring. When brood size is plotted against offspring size, it is clear that the largest offspring occurred in the smallest broods (<5 offspring). We also note that our laboratory-reared *G. holbrooki* had smaller broods than the wild-caught females, making an integer effect less likely for the wild-caught females. To explore this idea, we re-analyzed the offspring size data after removing mothers who produced three or fewer offspring. Without those very small broods in the analysis, there is no interaction between offspring number and the size class of mothers affecting offspring size (ANCOVA: *F*_1, 123_ = 0.09, *P *=* *0.77). This suggests that the stronger trade-off between offspring size and number exhibited by smaller females is driven by integer effects.

### Relationship between maternal size/age and offspring number

Larger mothers had more offspring in both the wild and in laboratory-reared females. Greater fecundity among larger mothers is seen in most taxa (meta-analysis: Lim et al. 2014) and has been repeatedly demonstrated in poeciliid fish, including *G. holbrooki* (Edwards et al. 2006, 2010; Benejam et al. 2009). Furthermore, when we experimentally disentangled the separate effects of maternal size and age we found no effect of age on fecundity. This result is contrary to the “cost of reproduction hypothesis” arising from life-history theory (Williams 1966; Skibiel et al. 2013). If senescence occurs then older mothers, who have a lower expectation of future reproductive success, are predicted to invest more in the present (e.g., terminal investment; Clutton-Brock 1984; Reznick 1985). In species such as *G. holbrooki* that show no postnatal parental care, this increased investment could only be mediated by an increase in offspring size and/or number. It follows from the optimality model for offspring size (Smith and Fretwell 1974) that increased investment should elevate fecundity. However, we did not see an age-mediated increase in offspring number, as reported in other species (Berteaux and Boutin 2000; Curtis Creighton et al. 2009), including *G. holbrooki* (Billman and Belk 2014). Possible explanations for the absence of an age effect are discussed below (see Study limitations).

### Relationship between offspring number and offspring size

The largest mothers in the wild appeared to mask a trade-off between offspring number and size. An offspring size-fecundity trade-off must occur at the individual level because mothers only have finite resources to allocate toward offspring (Brown 2003). This relationship is often not detected at the population level because some mothers are “resource rich” and invest more in both traits (van Noordwijk and de Jong 1986). When maternal size was not accounted for our wild-caught fish showed no relationship between offspring number and size, as reported in two other studies on *Gambusia*: in feral Australian populations of *G. holbrooki* (Trendall 1982) and native American populations of *G. affinis* (Swenton and Kodric-Brown 2012). Once the largest mothers were removed from the analysis, however, offspring size and number were negatively correlated, as reported in many other poeciliid fish (Abney and Rakocinski 2004; Swenton and Kodric-Brown 2012). This change in the relationship between offspring size and number suggests that the largest mothers were able to invest more in both offspring size and number due to their greater resource status (van Noordwijk and de Jong 1986; Christians 2000), thereby obscuring the more general trade-off.

### Study limitations

By experimentally generating large and small mothers of comparable age classes, we accumulated a number of confounding variables. The density, rearing temperature, and diet that our mothers experienced over the course of the experiment all differed between our treatment groups. Therefore, as is the case for wild *G. holbrooki*, the size and age of fish encompassed variation in the life history of the individuals. We cannot confirm that the apparent effects of maternal size were not due to an indirect effect of one of these confounding variables rather than a direct effect of maternal size. It is also possible that the slower growth environment experienced by older mothers may have masked an effect of age on fecundity. However, unless the effect of age exactly countered the effect of rearing environment, the fact that we observed no difference in offspring traits between old and young mothers of comparable size classes suggests that the additional 2 months that the older fish spent in the slow-growing conditions did not affect offspring traits. Another limitation of our study is that the age difference we generated between our old and young cohort (2 months) might have been insufficient to detect any effect of age on offspring traits. These limitations emphasize the difficulty of disentangling correlated variables: Independently manipulating age and size requires different rearing conditions, and if future studies seek to increase age difference they will also need to increase differences in rearing conditions to control for size. Future studies might do this using range of factors, each applied singly, so that they can identify whether it is maternal size per se or specific rearing conditions that generate offspring size differences.

## Conclusion

We investigated the relationship between maternal size and offspring size in a wild population of *G. holbrooki*, and experimentally tested for effects of maternal size and age on offspring size and number. As predicted, maternal size was positively correlated with both offspring number and size in the wild, consistent with the general pattern seen in other species. This trend was, however, driven by very large mothers. The experimental results were unexpected. Larger mothers had higher fecundity, but smaller offspring, possibly due to integer effects arising in small broods. These effects seem to be independent of maternal age, at least in the laboratory. Unfortunately, it remains unclear whether the size or age of very large mothers drives the positive correlation between maternal and offspring size observed in the wild. Future experiments need to take into account the possibility that there are nonlinear relationships between life-history traits that influence maternal allocation toward offspring.

## Data Accessibility

Data deposited at Dryad: doi:10.5061/dryad.46r30.
